# Protein phosphatase 1 suppresses androgen receptor ubiquitylation and degradation

**DOI:** 10.18632/oncotarget.6434

**Published:** 2015-11-30

**Authors:** Xiaming Liu, Weiwei Han, Sarah Gulla, Nicholas I. Simon, Yanfei Gao, Changmeng Cai, Hongmei Yang, Xiaoping Zhang, Jihong Liu, Steven P. Balk, Shaoyong Chen

**Affiliations:** ^1^ Hematology-Oncology Division, Department of Medicine, Beth Israel Deaconess Medical Center and Harvard Medical School, Boston, Massachusetts 02215, USA; ^2^ Department of Urology, Union Hospital, Tongji Medical School, Huazhong University of Science and Technology, Wuhan 430022, China; ^3^ Department of Pathogen Biology, Tongji Medical School, Huazhong University of Science and Technology, Wuhan 430030, China; ^4^ Department of Urology, Tongji Hospital, Huazhong University of Science and Technology, Wuhan 430030, China

**Keywords:** androgen receptor, prostate cancer, protein phosphatase 1

## Abstract

The phosphoprotein phosphatases are emerging as important androgen receptor (AR) regulators in prostate cancer (PCa). We reported previously that the protein phosphatase 1 catalytic subunit (PP1α) can enhance AR activity by dephosphorylating a site in the AR hinge region (Ser650) and thereby decrease AR nuclear export. In this study we show that PP1α increases the expression of wildtype as well as an S650A mutant AR, indicating that it is acting through one or more additional mechanisms. We next show that PP1α binds primarily to the AR ligand binding domain and decreases its ubiquitylation and degradation. Moreover, we find that the PP1α inhibitor tautomycin increases phosphorylation of AR ubiquitin ligases including SKP2 and MDM2 at sites that enhance their activity, providing a mechanism by which PP1α may suppress AR degradation. Significantly, the tautomycin mediated decrease in AR expression was most pronounced at low androgen levels or in the presence of the AR antagonist enzalutamide. Consistent with this finding, the sensitivity of LNCaP and C4–2 PCa cells to tautomycin, as assessed by PSA synthesis and proliferation, was enhanced at low androgen levels or by treatment with enzalutamide. Together these results indicate that PP1α may contribute to stabilizing AR protein after androgen deprivation therapies, and that targeting PP1α or the AR-PP1α interaction may be effective in castration-resistant prostate cancer (CRPC).

## INTRODUCTION

The androgen receptor (AR) plays a central role in prostate cancer (PCa) development and progression. Most patients initially respond to androgen deprivation therapy (ADT, surgical or medical castration), but the tumors inevitably recur despite castrate levels of androgen (castration-resistant prostate cancer, CRPC). AR expression is generally increased in CRPC, and one mechanism driving its activity is intratumoral synthesis of androgens from adrenal gland derived precursors or *de novo* from cholesterol [1–4]. This activity can be suppressed by drugs including abiraterone (which inhibits the enzyme CYP17A1 required for androgen synthesis) or by the direct AR antagonist enzalutamide, and both abiraterone and enzalutamide are now approved for treatment of CRPC [5, 6]. However, patients who respond to these agents generally relapse within a year, and AR appears to still be contributing to the growth of these relapsed tumors [7, 8]. Therefore, there remains a critical need to identify further mechanisms contributing to AR activity.

AR mRNA is highly expressed in CRPC with the AR gene being amplified in many cases [9, 10], while epigenetic mechanisms can further enhance AR gene transcription [11, 12]. AR activity in CRPC also may be enhanced by multiple kinase signaling pathways that directly or indirectly increase AR protein stability or transcriptional activity at low androgen levels [13, 14]. Recent findings have also underscored the significance of protein phosphatases in regulating AR and in PCa development. Protein phosphatase 2A (PP2A) can bind to AR and suppress its activity by dephosphorylation of several sites [15, 16]. The physiological relevance of PP2A in PCa development is supported by the identification of alterations in PP2A and its subunits in PCa model systems and clinical specimens during tumor progression [17–19]. In contrast to PP2A, we reported that the protein phosphatase 1 catalytic subunit (PP1α) can enhance AR activity by dephosphorylating a site in the hinge region, Ser650 [16]. Phosphorylation of this site was shown previously to enhance AR nuclear export [20], and we found that PP1α inhibition decreased nuclear expression of wild-type AR, but not an S650A mutant AR [16].

In this study we show that PP1α can also increase AR expression independently of S650 dephosphorylation. We find that PP1α binds to the AR ligand binding domain and decreases AR ubiquitination and degradation, particularly at low androgen levels or in the presence of AR antagonists. Mechanistically, we show that PP1α can dephosphorylate and inactivate ubiquitin ligases that target AR for ubiquitylation and degradation. Together these findings show that PP1α can contribute to maintaining AR protein expression and activity in CRPC, and that targeting PP1α or the AR-PP1α interaction may be a novel therapeutic approach.

## RESULTS

### PP1α can enhance AR activity independently of S650 phosphorylation site

**Figure 1:**
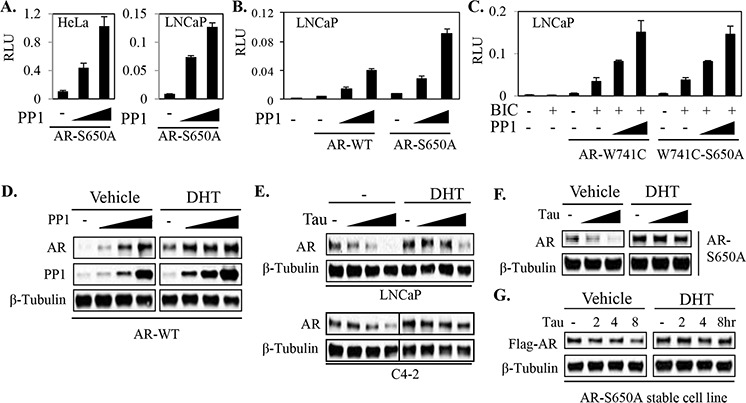
PP1α can stabilize and activate AR independently of phospho-S650 dephosphorylation **A.** HeLa and LNCaP cells were co-transfected with AR-S650A mutant and PP1α, together with ARE4-Luc reporter and CMV-Renilla (internal control). The cells were then incubated for overnight in androgen-depleted medium with DHT (10 nM) for dual-Luc analyses. **B.** LNCaP cells were co-transfected with Renilla (internal control) and ARE4-Luc reporters, together without or with AR wild-type (WT) versus AR-S650A mutant and PP1 catalytic subunit (PP1α). The cells were treated for overnight with androgens (10 nM of DHT) for Dual-Luc analysis. **C.** LNCaP cells in androgen-depleted medium were co-transfected with Renilla and ARE4-Luc reporters, together with AR-W741C construct versus AR-W741C-S650A mutant and PP1α as indicated. The cells were then incubated for overnight in androgen-depleted medium with bicalutamide (BIC, 10 μM) as indicated for Dual-Luc analysis. **D.** HeLa cells were transfected with Flag-PP1α and HA-AR and then incubated for overnight in androgen-depleted medium without or with androgen (10 nM of DHT) as indicated for blotting. **E.** LNCaP and C4–2 cells in androgen-depleted medium were treated overnight with tautomycin (Tau, 100, 200, and 400 nM) and androgen (10 nM DHT for LNCaP and 1 nM DHT for C4–2) as indicated and proteins were normalized for blotting. **F, G.** HeLa cells were transfected with AR S650A mutant (F) and a LNCaP line was generated to stably express Flag-tagged AR-S650A construct (G) Cells in androgen-depleted medium were treated for indicated time points with androgen (10 nM of DHT) and tautomycin (Tau, 400 nM) and proteins were normalized for blotting.

Phosphorylation of AR at S650 enhances its nuclear export and subsequent degradation [20]. We reported previously that PP1α interacts with AR and dephosphorylates S650, thereby increasing nuclear AR in PCa cells [16]. In support of this conclusion, we showed that PP1α inhibition with tautomycin decreased nuclear levels of the wild-type AR, but not an S650A mutant AR. However, in further studies we have found that PP1α overexpression enhances the transcriptional activity of both the wild-type and S650A mutant AR, as assessed by co-transfection of AR, PP1α and an AR regulated reporter gene into HeLa cells or LNCaP PCa cells (Figure 1A). Moreover, the effects of PP1α on the wild-type and S650A mutant AR were comparable, with the S650A having slightly more basal activity in the absence of cotransfected PP1α (Figure 1B). To discount effects from endogenous AR in LNCaP cells, we also examined the role of S650 in a W741C mutant AR, for which bicalutamide acts as an agonist versus being an antagonist for wild-type AR [21]. Significantly, the bicalutamide stimulated activities of both the W741C and W741C/S650A double mutant ARs in LNCaP cells were similarly increased by PP1α co-transfection (Figure 1C).

Together these results indicated that PP1α may regulate AR activity by one or more additional S650 independent mechanisms. Consistent with our previous report [16], PP1α overexpression in transient transfections increases AR protein (Figure 1D), and this increase could be prevented by treatment with the PP1α inhibitor tautomycin (Supplementary Figure S1). While tautomycin at comparable concentrations can also inhibit PP2A, we showed previously that PP2A increases AR degradation and that the PP2A-specific inhibitor fostriecin increases AR protein [16] (see also Supplementary Figure 2). PP1α inhibition with tautomycin also decreased endogenous AR protein in both LNCaP and C4–2 cells, particularly in steroid-depleted medium without addition of androgen (dihydrotestosterone, DHT) (Figure 1E). Significantly, tautomycin similarly decreased expression of the transiently transfected S650A mutant AR in HeLa cells (Figure 1F), and the stably expressed S650A mutant AR in LNCaP cells (Figure 1G, and Supplementary Figure S3). In both cases this effect was most pronounced in steroid-depleted medium in the absence of exogenous DHT. These results show that one mechanism through which PP1α can increase AR activity independently of S650 dephosphorylation is by increasing AR protein.

### Consensus PP1α-interacting KVFF motif in AR DNA binding domain is not essential for binding

**Figure 2:**
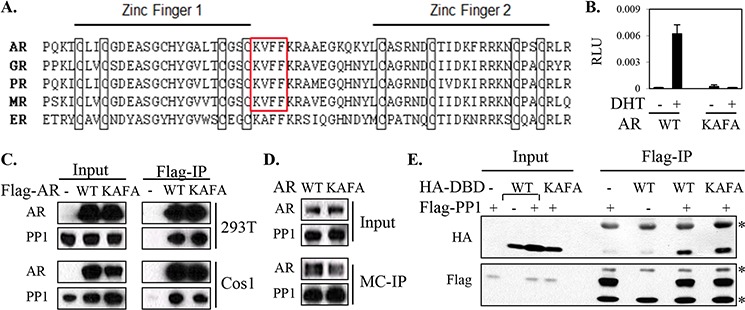
The potential PP1α-interacting KVFF motif of AR is not essential for AR-PP1α interaction **A.** A schematic drawing shows the linear amino acid sequence alignment of the zinc finger regions in the DNA binding domains of the class-I steroid receptors. Highlighted in the frame is the unique PP1α-interacting consensus motif (KVxF) that exists in AR, GR, PR, and MR, but not ERα. Amino acid sequences are based on human androgen receptor (AR; GenBank M20132.1); human glucocorticoid receptor (GRα; NM_001018077); human progesterone receptor (PR; M15716.1); human mineralocorticoid receptor (MR, NM_000901.3); and human estrogen receptor (ERα; NM_000125.3). **B.** AR-deficient PCa cell line (PC3) was transfected with AR-mediated probasin-Luc and CMV-Renilla reporters, together with AR wild-type (WT) versus KVFF mutant (KVFF to KAFA). Cells were then incubated for overnight in androgen-depleted medium with 10 nM of DHT for Dual-Luc analysis. **C.** 293T and Cos1 cells were co-transfected with PP1α and Flag-tagged AR wild-type versus KVFF mutant (KAFA), with the empty Flag vector (−) as control. Co-immunoprecipitation (Co-IP) assay was carried out using anti-Flag-M2 beads. **D.** HeLa cells transfected with AR wild-type versus KAFA mutant were incubated for overnight in androgen-depleted medium with 10 nM of DHT, followed by microcystin (MC)-IP and blotting. **E.** Flag-tagged PP1α was co-transfected in 293T cells with HA tagged AR-DBD wild-type (WT) versus DBD mutant (KAFA) constructs, followed by Co-IP using anti-Flag-M2 beads and blotting. Flag or HA empty vectors (−) were used as controls, respectively. Non-specific IgG bands are marked (*).

As PP1α and AR can form a complex [16], we next focused on the identification of molecular determinants mediating the PP1α-AR interaction and their role in increasing AR protein. PP1α has two consensus docking motifs shared by interacting partners, R/K-x(0,1)-V-x-F and F-x-x-R/K-x-R/K, where x stands for any amino acid [22]. AR has one potential PP1α binding motif, KVFF, locating in the DNA-binding domain (DBD) and in the vicinity of the zinc finger 1 that mediates DNA binding (Figure 2A). Interestingly, all class I steroid receptors (AR/GR/PR/MR) except ERα possess this motif. Moreover, we found that PP1α cotransfection could also enhance the transcriptional activity of GR and MR, but not ERα, suggesting a possible role for this site in mediating an interaction with PP1α (Supplementary Figure S4). Consistent with the essential function of this site for DNA binding, disruption of this motif by mutagenesis (KVFF to KAFA) resulted in complete transcriptional inactivation of AR (Figure 2B).

We then took a coimmunoprecipitation approach to assess for a role of this candidate motif in the AR-PP1α interaction. As shown in figure 2C, wild-type and KAFA mutant AR pulled down comparable amounts of PP1α in both 293T and Cos1 cell lines. As a complementary approach, we also used microcystin-agarose beads to precipitate endogenous PP1α and to assess its interaction with AR [16]. The results showed that in HeLa cells PP1α associates both with the wild-type and the KAFA mutant AR (Figure 2D). To further substantiate the above findings, we next generated an AR-DBD fragment and its mutated counterpart (DBD-KAFA) and assessed binding to PP1α. The results indicated that PP1α may interact with the AR-DBD, but that this interaction was not mediated by the KVFF motif (Figure 2E). Together, these results indicated that the KVFF site plays minimal if any role in PP1α-AR interaction.

### PP1α interacts predominantly with the AR ligand-binding domain

**Figure 3:**
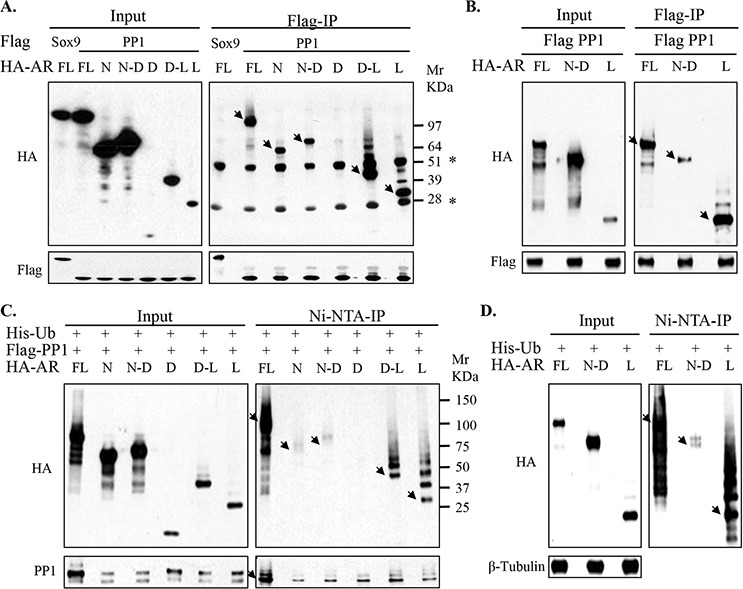
PP1α engages predominantly with the AR ligand-binding domain (LBD) that is enriched in ubiquitination **A.** 293T cells in medium containing 5% FBS were transfected with Flag-tagged PP1α (Flag-PP1α) and HA-tagged AR full length (HA-AR FL) versus additional AR constructs (N: NTD; N-D: NTD-DBD; D: DBD; D-L: DBD-LBD; and L: LBD), followed by anti-Flag co-IP analysis for blotting. Arrows indicate native AR bands and non-specific IgG bands are marked (*). Flag-tagged Sox9, which weakly interacts with AR, was used as control. **B.** Similarly, 293T cells were transfected with Flag-PP1α and HA-AR full length versus NTD-DBD and LBD constructs. After transfection, cells were then incubated for overnight in androgen-depleted medium for co-IP and blotting. **C.** 293T cells in androgen-depleted medium were transfected with His-Ub, Flag-PP1α, and HA-AR constructs as indicated, followed by Ni(nickel)-NTA-IP under denatured condition and blotted. **D.** Similarly, 293T cells in androgen-depleted medium were transfected with His-Ub and indicated HA-AR constructs, followed by Ni-NTA-IP under denatured conditions. Arrowheads in the upper panels mark positions of the native AR constructs and an arrowhead on the lower panel of (C) marks the mono-ubiquitylated PP1α. Mr: Molecular weight marker in Kilodalton (KDa).

We next systematically assessed the association between PP1α and a panel of constructs spanning the AR N-terminal domain (NTD) that bears the major transcriptional activation function, the central DNA-binding domain (DBD), and the C-terminal ligand-binding domain (LBD). Analysis in androgen-containing medium demonstrated that PP1α could associate with the isolated NTD and LBD, with the inclusion of the DBD not causing an appreciable increase in binding (Figure 3A). Comparison with the inputs further indicates that PP1α has higher affinity for the AR LBD than the NTD. We similarly observed preferential binding to the AR LBD when cells were cultured in steroid-depleted medium (Figure 3B). It should be noted while AR is primarily cytoplasmic in the absence of ligand and accumulates in the nucleus in response to androgen, PP1α is highly expressed in the nucleus and cytoplasm (Supplementary Figure S5). Therefore, cellular localization is less likely to be a major factor dictating the association between PP1α and these AR domains.

Notably, the PP1α-associated AR migrated on the gels as a major band with a series of distinct minor bands separated by ∼8 KDa, consistent with ubiquitylation. To confirm that these bands were ubiquitylated AR, we co-expressed histidine-tagged ubiquitin (his-Ub) together with PP1α and AR, and then used Nickel(Ni)-NTA resin under denaturing conditions to pull-down ubiquitylated proteins. Immunoblotting for the HA-tagged AR then revealed higher molecular weight bands indicative of ubiquitylation associated primarily with constructs containing the LBD (Figure 3C). It should be noted that we also observed bands whose migration was consistent with the unmodified DBD-LBD or LBD fragments (see also Supplementary Figure S6), which we presume reflect weak nonspecific binding to the resin compared to the ubiquitylated proteins that were highly enriched by the resin. We observed a similar pattern in the absence of transfected PP1α, consistent with the AR LBD being the primary target for ubiquitylation (Figure 3D).

### PP1α stabilizes AR by decreasing AR LBD polyubiquitylation

**Figure 4:**
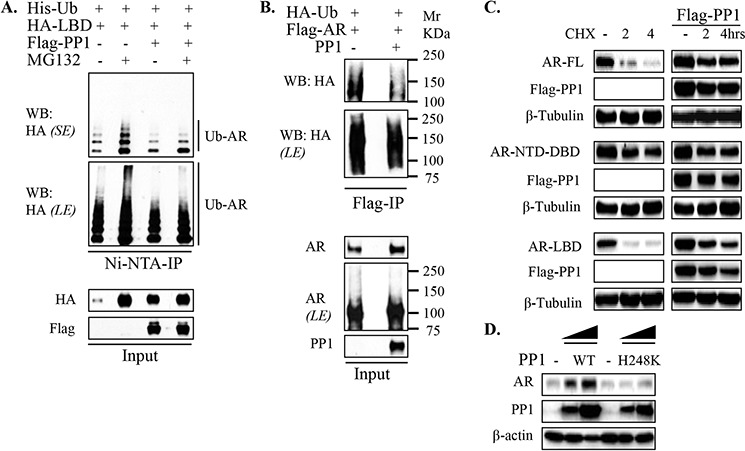
PP1α stabilizes AR by attenuating LBD-directed AR ubiquitylation **A.** 293T cells transfected with His-Ub, HA-AR-LBD and Flag-PP1α were incubated overnight in androgen-depleted medium without or with MG132 (10 μM) as indicated, followed by Ni-NTA-IP under denatured conditions and blotting. *SE*: short exposure; *LE*: long exposure. **B.** 293T cells were transfected with HA-Ub, Flag-AR-full-length together without or with PP1α. Cells were then incubated overnight in androgen-depleted medium, followed by anti-Flag-IP under denatured conditions. Mr: Molecular weight marker in Kilodalton (KDa). **C.** HeLa cells in androgen-depleted medium were co-transfected with HA-AR full-length (FL), NTD-DBD and LBD constructs, without or with Flag-PP1α and cycloheximide as indicated. Total proteins were harvested and normalized for blotting. **D.** HeLa cells in androgen-depleted medium were co-transfected with AR and PP1α wild-type versus its enzymatically inactive counterpart (H248K). Cells were then incubated in androgen-depleted medium for blotting.

The above findings suggested that PP1α may stabilize AR protein by decreasing ubiquitylation of the LBD. To examine this further, we assessed effects of PP1α overexpression and proteasome inhibitor (MG132) treatment on ubiquitylation of the AR LBD. In the absence of transfected PP1α, treatment with MG132 increased both the levels of oligo- and poly-ubiquitylated AR LBD (Figure 4A). In contrast, these ubiquitylated AR species, and in particular the polyubiquitylated AR, were not increased by MG132 in cells cotransfected with PP1α. Cotransfection of PP1α similarly decreased the polyubiquitylation of full length AR (Figure 4B).

To determine whether this PP1α mediated decrease in AR polyubiquitylation resulted in decreased AR degradation, we next used cycloheximide to block new protein synthesis and thereby assess AR protein stability. PP1α cotransfection markedly decreased degradation of full length AR (Figure 4C). Moreover, consistent with PP1α suppressing polyubiquitylation of the AR LBD, PP1α cotransfection also markedly increased stability of the AR LBD, but not the AR NTD-DBD fragment (Figure 4C). Finally, PP1α with an H248K mutation that inactivates its phosphatase activity was unable to increase AR protein, indicating that PP1α stabilizes AR through dephosphorylation of one or more substrates (Figure 4D).

### PP1α dephosphorylates ubiquitin ligases mediating AR degradation

Several ubiquitin ligases, including MDM2 and SKP2, have been implicated as mediators of AR ubiquitylation and degradation [23–26]. MDM2 degradation can be enhanced by phosphorylation at multiple sites including S395, and previous reports indicate that PP1α can decrease the degradation of MDM2 by dephosphorylating this site [27]. However, MDM2 activity can also be enhanced by growth factor stimulated phosphorylation of sites including S166 and S186 [28]. Effects of PP1α on SKP2 have not been addressed previously. Therefore, we next assessed whether PP1α may dephosphorylate and inactivate these ubiquitin ligases to attenuate AR ubiquitylation and degradation.

**Figure 5:**
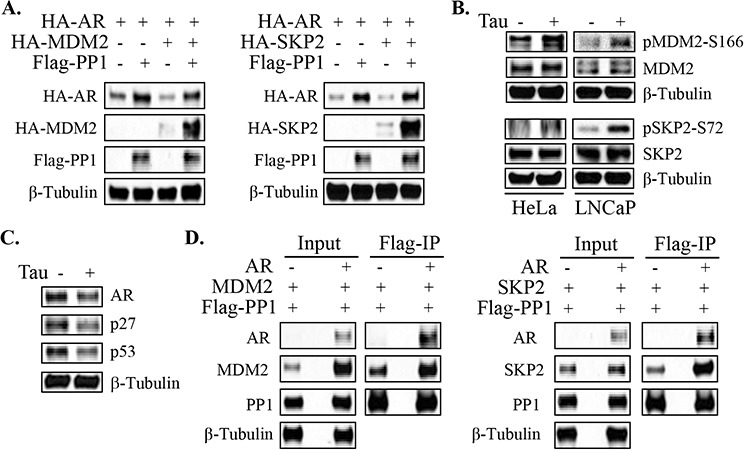
PP1α can dephosphorylate AR degrading E3 ligases **A.** HeLa cells were co-transfected with HA-AR, Flag-PP1α and HA-tagged MDM2 or SKP2 as indicated. Cells were then incubated for overnight in androgen-depleted medium for blotting. **B.** HeLa and LNCaP cells in androgen-depleted medium were treated for 3 hrs with 200 nM of tautomycin, followed by protein normalization for blotting. **C.** LNCaP cells in androgen-depleted medium were treated for 3 hrs with tautomycin as indicated. Total proteins were normalized for blotting. **D.** HeLa cells were transfected of Flag-PP1 and HA-MDM2 (left panel) or SKP2 (right panel), without or with AR as indicated. Cells were then incubated for overnight in androgen-depleted medium, followed by anti-Flag Co-IP and blotting.

Consistent with previous reports, AR protein expression was decreased by cotransfection of MDM2 or SKP2 (Figure 5A). Cotransfection of PP1α resulted in a dramatic increase in the levels of transfected MDM2 and SKP2, which appears to be primarily due to increased transcription and translation from the expression vectors rather than decreased protein degradation. However, this may also in part reflect PP1α mediated inactivation of MDM2 and SKP2 enzymatic activities, as this would suppress autoubiquitylation and degradation [29, 30]. In either case, despite the dramatic increases in MDM2 and SKP2, there were no increases in the ability of these proteins to suppress AR expression, indicating that their activity was markedly impaired by PP1α (Figure 5A).

To determine whether endogenous PP1α may regulate the phosphorylation and activity of these ubiquitin ligases, we treated HeLa and LNCaP cells with tautomycin and assessed for effects on endogenous MDM2 and SKP2. We did not observe clear increases in MDM2 or SKP2 in either cell line (Figure 5B). However, tautomycin increased MDM2 phosphorylation at S166, which has been reported to enhance MDM2 activity [28]. Similarly, tautomycin increased SKP2 phosphorylation at S72, a site that may be phosphorylated by AKT and lead to increased stability and activity [31, 32]. Consistent with increased activity of MDM2 and SKP2, tautomycin treatment decreased levels of their respective substrates, p53 and p27 (Figure 5C).

Taken together these findings indicated that PP1α inhibition may increase AR ubiquitylation and degradation by increasing the activity of one or more AR degrading ubiquitin ligases. However, it was not clear whether or how these effects on MDM2 of SKP2 were related to the PP1α-AR interaction. Therefore, we next asked whether AR could function as a scaffold to bridge PP1α and MDM2 or SKP2. HeLa cells were transfected with Flag-tagged PP1α and MDM2 or SKP2, with or without cotransfected AR. Anti-Flag immunoprecipitation then indicated that the PP1α-SKP2 association was substantially increased by cotransfected AR, consistent with a scaffold function (Figure 5D). AR cotransfection similarly increased the level of MDM2 that was coprecipitated with PP1α, but AR also increased input levels of MDM2, making interpretation of the result less clear (Figure 5D). Further studies to address this issue are now focused on identification of site directed mutations that can selectively impair the AR-PP1α interaction.

### Androgen deprivation and AR antagonists sensitize PCa cells to PP1α inhibition

**Figure 6:**
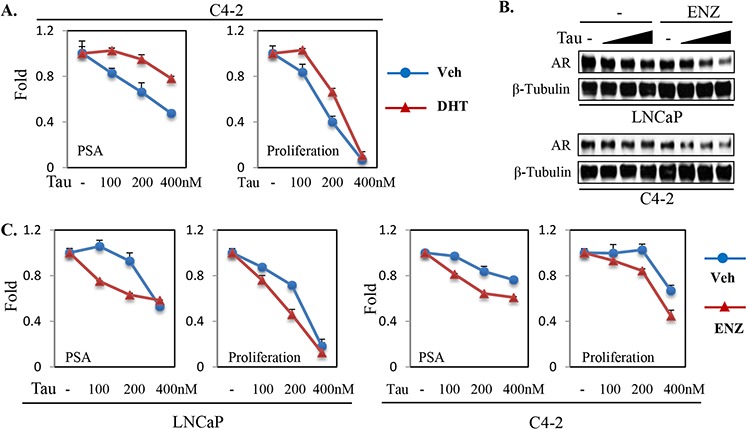
Androgen ablation increases PP1α-dependence for AR protein expression and transactivation in PCa cells **A.** C4–2 cells in androgen-depleted medium were treated with tautomycin and androgen (1nM DHT) for qRT-PCR analysis of PSA expression (24 hr treatment) or for cell proliferation (3-day treatment), with the results being normalized to the no tautomycin levels in the absence and presence of DHT. Veh: vehicle; DHT: 1 nM of DHT. **B, C.** LNCaP and C4–2 cells in androgen-containing medium (10% FBS) were treated with enzalutamide (ENZ, 10 μM) and tautomycin (100, 200, and 400 nM) as indicated. Total proteins (for 24 hr treatment) were harvested and normalized for blotting (B) and total RNA (for 24 hr treatment) was isolated for qRT-PCR analysis of PSA expression (C). The cell proliferation analysis was carried out after 3-day treatment (C). The gene expression and cell growth results were normalized to no tautomycin in the absence and presence of enzalutamide (C). Veh: vehicle; Enz: 10 μM of enzalutamide.

As noted in Figure 1, effects of tautomycin on AR protein expression in LNCaP and C4–2 cells were most marked under conditions of androgen deprivation. Therefore, we compared the effects of tautomycin in C4–2 cells cultured in androgen depleted medium without or with added DHT. Consistent with its effects on AR protein under these conditions, tautomycin was more potent at reducing expression of PSA and proliferation under androgen depleted conditions (Figure 6A).

Significantly, effects of tautomycin on AR protein expression in LNCaP and C4–2 cells were also enhanced by treatment with the AR antagonist enzalutamide (Figure 6B). Therefore, we examined the effects of tautomycin on AR activity in LNCaP and C4–2 cells without and with enzalutamide. In both cell lines, the inhibitory effects of tautomycin were enhanced by enzalutamide (Figure 6C). Collectively these findings indicate that agents targeting PP1α may enhance the efficacy of androgen deprivation therapies and be effective in CRPC.

## DISCUSSION

AR undergoes basal and ligand stimulated phosphorylation at multiple sites, and these modifications can modulate it stability, cellular localization and transcriptional activity. AR phosphorylation may also be regulated by protein phosphatases, including PP2A and PP1. PP2A can dephosphorylate multiple sites in the AR N-terminal domain, including S81, S94, S258, S308, and S424, that are normally increased in response to androgen and are associated with increased AR transcriptional activity [15]. Significantly, PP2A access to these sites may be negatively regulated by androgen binding, indicating that a physiological function of PP2A may be to suppress basal AR activation in the absence of androgen [33].

In contrast to PP2A, we had previously shown that PP1α could bind to AR and dephosphorylate AR at S650 in the hinge region and thereby suppress AR nuclear export, providing a mechanism by which PP1α can enhance AR activity [7]. In the current study we found that PP1α could also suppress the polyubiquitylation of the AR LBD and its subsequent degradation, and that PP1α inhibition with tautomycin could thereby decrease AR expression independently of S650 dephosphorylation. Mechanistically, we found that PP1α inhibition with tautomycin increased the phosphorylation of MDM2 and SKP2 at sites that could enhance their activities, suggesting that PP1α may suppress AR ubiquitylation by dephosphorylating these sites. Further results indicated that AR may mediate an interaction between PP1α and these ubiquitin ligases, but further studies are needed to determine the relationship between the AR-PP1α association and PP1α mediated dephosphorylation of MDM2, SKP2, and potentially other ubiquitin ligases.

This study also found that PP1α binding to AR is mediated primarily by the AR LBD. Although the precise site on the LBD remains to be determined, it does not appear to be the coactivator binding site as binding is not ligand dependent. We also cannot rule out that binding is indirect and mediated by a not yet identified PP1 regulatory protein. An attractive hypothesis is that PP1α associated with the AR LBD directly protects AR from ubiquitylation by locally inactivating ubiquitin ligases. Alternatively, PP1α may more globally suppress a subset of ubiquitin ligases, with the AR associated PP1α having distinct functions including the dephosphorylation of S650. Further studies are underway to test these hypotheses.

In any case, these findings indicate that therapies targeting PP1α, or possibly the AR-PP1α interaction, may have efficacy in CRPC. Significantly, the decrease in AR protein in response to tautomycin was most dramatic under androgen depleted conditions and in cells treated with the AR antagonist enzalutamide. Therefore, PP1α activity may play a particularly important role in maintaining AR expression in CRPC and in resistance to enzalutamide.

## MATERIALS AND METHODS

### Materials

Anti-Flag M2 affinity gel was from Sigma-Aldrich, tautomycin and fostriecin were from Calbiochem and Enzo Life Science, Ni-NTA agarose was from Qiagen, and microcystin-agarose was from EMD Millipore. Anti-AR, anti-PP1, anti-HA, anti-p27, and anti-p53 antibodies were from Cell Signaling, anti-β-Actin was from Abcam, anti-β-tubulin was from EMD Millipore, and anti-flag-M2 was from Sigma-Aldrich. The anti-SKP2 (Santa Cruz) and anti-p-SKP2-S72 (Cell Signaling) antibodies were kindly provided by Dr. Inuzuka Hiroyuki, and the anti-MDM2 and anti-p-MDM2-S166 (Santa Cruz) antibodies were kindly provided by Dr. Ming Chen (BIDMC, Harvard Medical School, Boston, USA). FBS and CDS (charcoal-dextran stripped serum) were from Hyclone.

### Immunoprecipitations (IPs)

For coimmunoprecipitation (co-IP), cells were harvested in triton lysis buffer (TLB) containing protease inhibitors. The lysates were incubated on ice for 20 min and centrifuged for 5 min at maximum speed. 20 μl of the supernatant was saved as input and the rest was incubated with anti-Flag beads, followed by incubation at 4°C on a rotator for 2 hr. Then the input and washed (4x) IP beads were boiled in 60 μl of Laemmli buffer containing 5% β-mercaptoethanol. For immunoprecipitation (IP) under denatured conditions, cells were harvested by boiling in 2% SDS followed by 20x dilution the TLB. The test then proceeded as the above regular IP. For the ubiquitylation and Ni(nickel)-NTA-IP assay, the cells were washed in PBS and an aliquot was lysed in RIPA buffer as input. The rest was lysed in buffer A (6M guanidine HCl, 0.1 M Na2HPO4/NaH2PO4, 10 mM imidazole, pH 8.0), followed by sonication and centrifugation. The supernatants were incubated with Ni-NTA beads at room temperature for 3 hrs, followed by wash 2x with buffer A, 2x with buffer A/buffer TI (1:3), and 1x with buffer TI (25 mM Tris-Cl, 20 mM imidazole, pH 6.8). Then both input and the IP yields were boiled in 60 μl of Laemmli buffer containing 5% β-mercaptoethanol. Microcystin-IP was as previously described [16].

### Gene expression analysis

RNA isolation was carried out using the TriZOL reagent and the qRT-PCR analysis on gene expression was performed with the TaqMan One-Step RT-PCR Master Mix Reagents (Applied Biosystems). The TaqMan primer and probe sets for gene expression assay: PSA: Forward, 5′- GATGAAACAGGCTGTGCCG-3′; Reverse, 5′- CCTCACAGCTACCCACTGCA-3′; Probe, 5′- FAM-CAGGAACAAAAGCGTGATCTTGCTGGG-3′. The TaqMan primer-probe set for GAPDH transcripts (internal control) was purchased as inventoried mix from Applied Biosystems.

### Cell culture, cellular fractionation, transient transfection and reporter gene assays

LNCaP cells were grown in the RPMI-1640 medium and C4–2 and VCaP cells in DMEM medium containing 10% FBS. PC3, 293T, HeLa, and Cos1 cells were grown in DMEM medium with 5% FBS. For androgen-starved conditions, cells were grown in medium containing 5% CDS. Cellular fractionation assay were carried out with the NE-PER kit (Pierce), following the manufacture's direction. For transfection, cells were grown in normal growth medium to ∼80% confluence and plasmid DNA was transfected with LipofectAMINE 2000. Empty relevant vectors (such as pCDNA3.1, HA vector, and Flag vector) were supplemented to equalize the total amount of plasmids in the transfections. After overnight transfection, the cultures were refreshed with indicated medium for treatments. The pCMV-pRL-Renilla and androgen-responsive reporter plasmids have been described previously [16]. Additional steroids, steroids receptors and reporters were from the Balk lab. Luciferase activities were measured with the Dual-Luc assay kit (Promega). The ratios between Firefly and Renilla luciferase activities are shown as relative light units (RLU), and the results reflect the mean and standard deviation from triplicate samples.

### Cell proliferation assay

Cells were cultured in 96-well plates for 2 days and then treated for 3 days as indicated. The cell proliferation analysis was performed with the CellTiter-Glo assay kit (Promega), following the manufacture's manual.

### Western blot data processing

The Western blotting was developed based on X-Ray film (Research Products International) and the western lightning Plus-ECL reagent (PerkinElmer). The images were acquired using the CanoScan LiDE 210 scanner and processed using the Adobe Photoshop 7.0 software. Relevant images were quantified by using the ImageJ software. All gels shown are representative of results from at least three experiments.

### Plasmids and DNA mutagenesis

The AR constructs were generated based on the pcDNA3.1(+)-Kozak-HA-HA vector and cover the following regions: HA-HA-AR full-length (aa 1–919); HA-HA-AR-NTD (aa 1–539); HA-HA-AR-NTD-DBD (aa 1–628); HA-HA-AR-DBD (aa 538–628); and HA-HA-AR-DBD-LBD (aa 538–919). The HA-AR-LBD (aa 662–919) vector was from our Lab. The untagged PP1α plasmid was from Origene and then cloned into the 3x-Flag-CMV-10 vector (Sigma-Aldrich) to generate the Flag-PP1α expression plasmid. The his-Ub, Flag-Ub, Flag-Sox9, HA-MDM2 and HA-SKP2 expression plasmids were kindly provided by Drs. Pengda Liu, Wenjian Gan, Xin Yuan, and Inuzuka Hiroyuki (BIDMC), respectively. DNA mutagenesis on AR and PP1α was performed with the Quick-change site-directed mutagenesis Kit (Stratagene). Details for plasmids and mutants generation are available upon request.

## SUPPLEMENTARY FIGURES LEGENDS


