# Supplementary material for: Protein phosphatase 1 suppresses androgen receptor ubiquitylation and degradation
Source: Oncotarget. 2015 Nov 30;7(2):1754–64. doi: 10.18632/oncotarget.6434 (PMC4811495; doi:10.18632/oncotarget.6434)
Supplement: Supplementary file 1 [file oncotarget-07-1754-s001.pdf]

SUPPLEMENTARY FIGURES LEGENDS

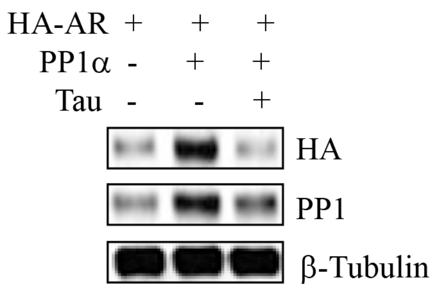

Supplementary Figure S1: PP1 inhibitor tautomycin could reverse PP1-mediated stimulation of AR protein expression. HeLa cells in androgen-depleted medium were transfected with HA-AR and PP1 as indicated, followed by treatment without or with tautomycin (Tau, 200nM) for 4 hrs. Total proteins were normalized for blotting.

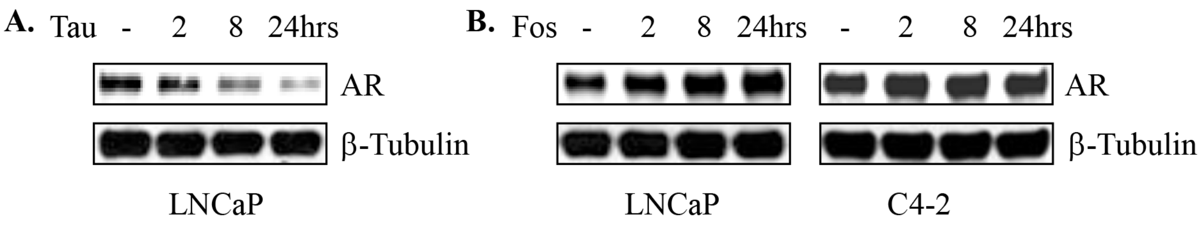

Supplementary Figure S2: PP1 inhibitor tautomycin decreased and PP2A-specific inhibitor fostriecin increased AR protein expression in prostate cancer cells. A. LNCaP cells in androgen-depleted medium were treated with tautomycin (Tau, 200nM) as indicated. Total proteins were normalized for blotting. **B.** LNCaP and C4–2 cells in androgen-depleted medium were treated with fostriecin (Fos, 100nM) as indicated. Total proteins were normalized for blotting.

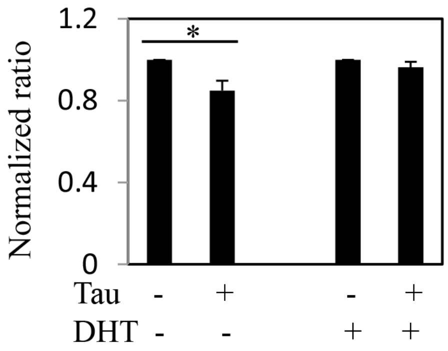

Supplementary Figure S3: Under androgen deprivation, PP1 inhibitor tautomycin decreased AR-S650A protein expression in LNCaP stable cells. LNCaP Flag-AR-S650A stable cell line in androgen-depleted medium was treated without or with androgen (10nM of DHT) for overnight, followed by treatment with tautomycin (Tau, 400nM) for 4 hrs. Total proteins were normalized for blotting and triplicated tests were quantified. The results represented the normalized ratio between the signals of Flag-AR and beta-tubulin, with the untreated samples set as 1. A star (*) marks significant difference based on the student's *t*-test value of less than 0.05 (0.00568 for the tests of no androgen versus 0.0804 for the tests with DHT).

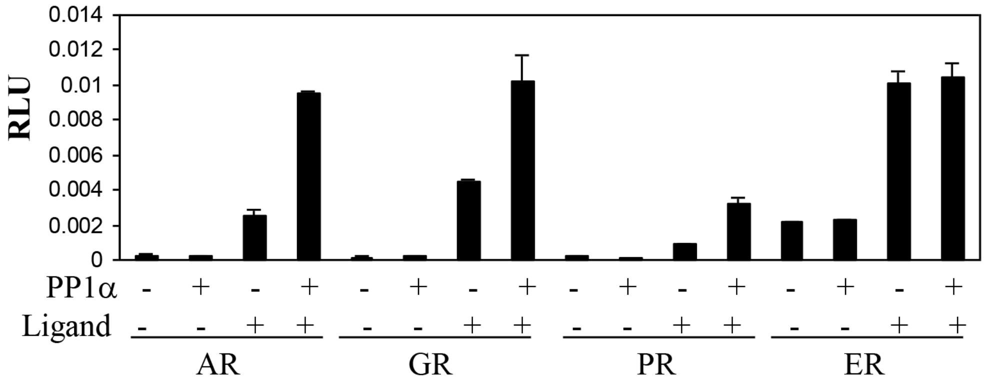

Supplementary Figure S4: PP1 stimulated transactivation of AR/GR/PR, but not ER. LNCaP cells in androgen-depleted medium were co-transfected of Renilla (internal control) and AR/GR/PR and MMTV-Luc versus ERα and ERE2-Luc reporters, together without or with PP1 as indicated. The cells were then treated for overnight without or with specific ligands (10nM of androgen for AR, 10 nM of dexamethasone for GR, 10nM of progesterone for PR, and 10nM of E2 for ERα) for Dual-Luc analysis.

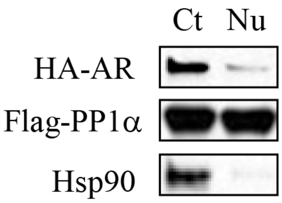

Supplementary Figure S5: Cellular distribution of PP1 and AR in the absence of androgen. 293T cells in androgen-depleted medium were co-transfected of Flag-PP1 and HA-AR wild-type, followed by cellular fractionation and blotting. Ct: cytoplasmic fraction; Nu: nuclear fraction. As a control, Hsp90 was blotted as the cytoplasmic marker

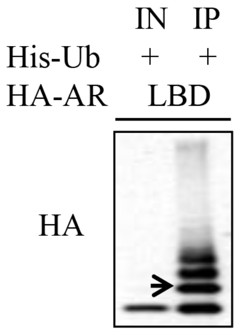

Supplementary Figure S6: AR LBD ubiquitination. 293T cells in androgen-depleted medium were transfected of his-tagged Ub, flag-tagged PP1α, and HA-tagged AR LBD constructs, followed by Ni(nickel)-NTA-IP analysis and blotting. An arrowhead indicates the monoubiquitinated AR LBD. IN: input; IP: NTA-IP
